# Challenging the Continued Usefulness of Social Media Recruitment for Surveys of Hidden Populations of People Who Use Opioids

**DOI:** 10.2196/63687

**Published:** 2025-04-30

**Authors:** Elizabeth D Nesoff, Joseph J Palamar, Qingyue Li, Wenqian Li, Silvia S Martins

**Affiliations:** 1 Department of Biostatistics, Epidemiology, and Informatics University of Pennsylvania Perelman School of Medicine Philadelphia, PA United States; 2 Department of Population Health NYU Langone Health New York, NY United States; 3 University of Pennsylvania School of Social Policy and Practice Philadelphia, PA United States; 4 Department of Epidemiology Columbia University Mailman School of Public Health New York, NY United States

**Keywords:** opioids, substance use, survey methods, social media, recruitment, survey, drug overdose, substance use disorder, online recruitment, online survey, mental health, addiction, data collection

## Abstract

Historically, recruiting research participants through social media facilitated access to people who use opioids, capturing a range of drug use behaviors. The current rapidly changing online landscape, however, casts doubt on social media’s continued usefulness for study recruitment. In this viewpoint paper, we assessed social media recruitment for people who use opioids and described challenges and potential solutions for effective recruitment. As part of a study on barriers to harm reduction health services, we recruited people who use opioids in New York City to complete a REDCap (Research Electronic Data Capture; Vanderbilt University) internet-based survey using Meta (Facebook and Instagram), X (formerly known as Twitter), Reddit, and Discord. Eligible participants must have reported using opioids (heroin, prescription opioids, or fentanyl) for nonprescription purposes in the past 90 days and live or work in New York City. Data collection took place from August 2023 to November 2023. Including study purpose, compensation, and inclusion criteria caused Meta’s social media platforms and X to flag our ads as “discriminatory” and “spreading false information.” Listing incentives increased bot traffic across all platforms despite bot prevention activities (eg, reCAPTCHA and counting items in an image). We instituted a rigorous post hoc data cleaning protocol (eg, investigating duplicate IP addresses, participants reporting use of a fictitious drug, invalid ZIP codes, and improbable drug use behaviors) to identify bot submissions and repeat participants. Participants received a US $20 gift card if still deemed eligible after post hoc data inspection. There were 2560 submissions, 93.2% (n=2387) of which were determined to be from bots or malicious responders. Of these, 23.9% (n=571) showed evidence of a duplicate IP or email address, 45.9% (n=1095) reported consuming a fictitious drug, 15.8% (n=378) provided an invalid ZIP code, and 9.4% (n=225) reported improbable drug use behaviors. The majority of responses deemed legitimate (n=173) were collected from Meta (n=79, 45.7%) and Reddit (n=48, 27.8%). X’s ads were the most expensive (US $1.96/click) and yielded the fewest participants (3 completed surveys). Social media recruitment of hidden populations is challenging but not impossible. Rigorous data collection protocols and post hoc data inspection are necessary to ensure the validity of findings. These methods may counter previous best practices for researching stigmatized behaviors.

## Introduction

Opioid-involved overdoses significantly increased across the United States through 2023 [1]. Drug overdose deaths more than doubled from 2015 to 2023—increasing from 53,356 to 107,543—and 78% of drug overdose deaths in 2023 involved opioids [2,3]. Since drug use is an illegal and heavily stigmatized behavior, most research conducted on nonprescription opioid use recruited people who use opioids through settings or organizations frequented by the target population, such as syringe exchange programs or substance use disorder treatment facilities [4,5]. However, these venue-based recruitment methods may introduce selection bias as certain racial or ethnic minorities and women may feel less comfortable in and around these spaces [6-10].

Internet-based recruitment may provide an alternative method for accessing certain hard-to-reach populations and stigmatized behaviors. The primary barrier to feasibility in internet-based recruitment is access to people who use opioids, given the highly stigmatized nature of drug use [4,5]. Many popular social media platforms [11-13] provide a sophisticated suite of tools to target advertisement placement to the feeds of specific audiences defined by demographic characteristics (eg, age and location) and contextual interests such as pages or groups to which subscribers indicated interest, keyword searches, and frequently used hashtags [11-15]. Different advertisement images could also be targeted to different groups based on race and ethnicity to increase participant diversity [16]. Systematic reviews found that, compared with traditional recruitment methods, social media recruitment had lower costs, shorter recruitment periods, and improved representation of populations with mental health and substance use problems [4,14,15]. Social media platforms have been shown to be effective for recruitment in studies of populations reporting addiction, mental health problems, and other hard-to-reach populations [11,12,14,15,17-19]. In addition, while some racial or ethnic minority groups and people of lower income have had less access to internet services historically [20], studies have also shown that social media recruitment effectively engages low-socioeconomic status and racial and ethnic minority participants [16,21-23].

However, the current, rapidly changing online landscape calls into question the continued usefulness of social media platforms for recruiting and surveying people who use opioids. In January 2022, Meta, which owns Facebook and Instagram, reduced their ad-targeting options for what it calls “sensitive” topics in an effort to improve user privacy [24]. Advertisers are no longer allowed to use custom keywords or hashtags to target subscribers but must fit their ads into prescribed categories within Meta’s ad management system. Twitter, now X, is in flux after the November 2022 acquisition by Elon Musk [25]. Starting in July 2023, X began limiting the number of tweets a user can read or send in a day depending on their subscription level, leading to decreased user engagement and subscriber loss [26,27]. Reddit is also somewhat volatile. As part of the leadup to Reddit’s March 2024 IPO, Reddit announced they will charge companies such as third-party web browsing apps that want to access its content using an application programming interface (API) [28]. The API fees were unpopular among Reddit users, who saw it as the company prioritizing profit over the community’s preferences [28]. Reddit fora, known as subreddits, are run by volunteer, unpaid moderators who enforce subreddit standards of conduct (eg, no posts about drug selling). Many subreddits shut down in protest for 2 weeks in June 2023, and in response, Reddit announced it intends to reduce the power of moderators [28,29]. These changes in user engagement and advertising options across social media platforms call into question the continued usability of social media for identifying and recruiting hard-to-reach populations for health research.

In addition, the proliferation of automated bots and malicious responders threatens internet-based survey research data integrity for incentivized surveys. Including a participation incentive increases the likelihood of attracting bots and malicious responses—a consideration that must be weighed against the effectiveness of incentives in increasing participation among the target population [30-32]. Human respondents easily create bots that bypass survey platforms’ safety protocols and data protection mechanisms or use virtual private servers to repeatedly complete surveys for financial gain [32,33]. While bots are thought to be the most prolific threat to data integrity, human threats to data validity for incentivized surveys are becoming more common and increasingly difficult to detect [31]. Individual fraudsters may falsify responses to mimic what they perceive as desirable survey answers in order to receive survey incentives [31,34]. “Click farms” groups that purposefully search for surveys with financial incentives may also contribute to invalid responses [31]. Scammers may take surveys multiple times, slightly altering their answers to appear like unique participants [31,34,35]. Even respondents who are from the target population may provide distorted or misleading survey responses if answers are rushed or careless or if they take a survey multiple times to receive a larger amount of the financial incentive [31,36].

The purpose of this Viewpoint is to share our experiences recruiting a hard-to-reach population through social media. As part of a study on barriers to engagement with harm reduction health services such as syringe exchange, we aimed to recruit people who use opioids in New York City to an internet-based survey. Eligible participants reported past-3-month use of opioids (including heroin, prescription opioids, fentanyl, and newer and emerging opioids like U-47700 and nitazenes) for nonprescription purposes (eg, to get “high”). Because the goal of our study was to investigate barriers to harm reduction engagement, we could not directly recruit from harm reduction health services organizations—a traditional point of access for studies of people who use opioids—as these people who use opioids would presumably experience lower engagement barriers. Recruiting participants through social media previously facilitated access to a group of people who use opioids traditionally not included in research and captured a greater range of drug use behaviors [4,15]. In this paper, we assess the continued usefulness of social media recruitment for this hidden population and describe challenges and potential solutions for effective recruitment across social media platforms.

## Methods

### Participant Recruitment

We recruited participants across 4 social media platforms: Meta (Facebook and Instagram), X (Twitter), Reddit, and Discord. Unless otherwise indicated, we tracked ad engagement (impressions and link clicks) and cost per click. Specific activities and challenges for each platform are described below, including the ad purchasing and approval processes.

#### Meta

Advertisements on Facebook and Instagram are distributed concurrently through Meta’s ad management platform. The user sets a campaign goal—in our case, increasing website traffic measured by link clicks—a daily spending limit and campaign duration. Meta’s algorithm for how cost per click is calculated lacks transparency, and it was not clear if link clicks consistently translated to completed surveys.

**Figure 1:**
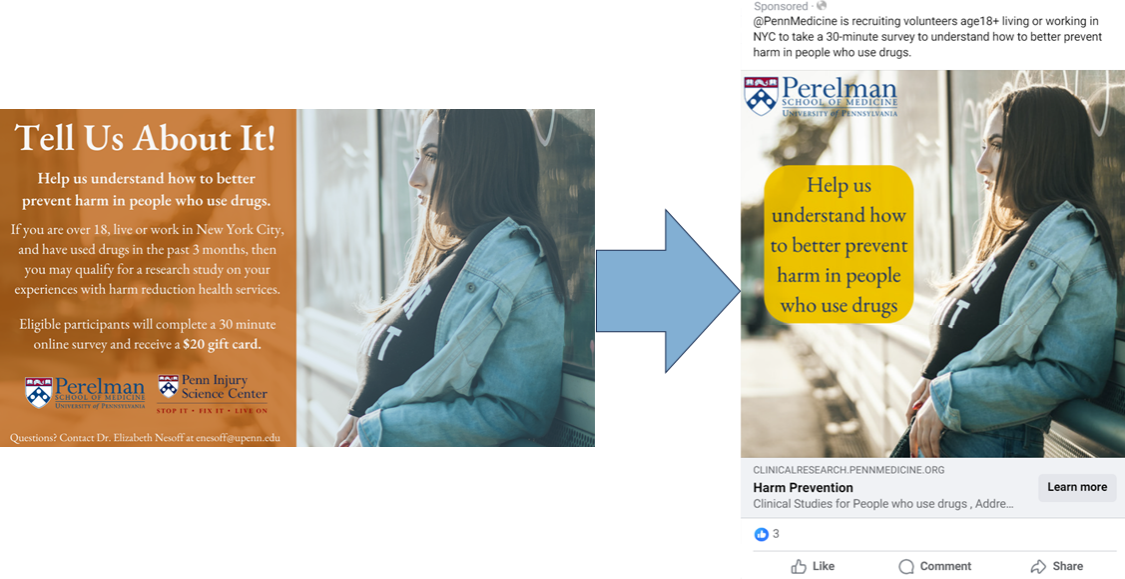
Example of an approved institutional review board (IRB) advertisement versus an approved Meta advertisement. NYC: New York City.

As previously stated, Meta no longer supports the use of custom keywords or hashtags to target subscribers. Meta’s prescribed ad categories did not provide options related to drug use, and ads with images of drug paraphernalia, drug use, or smoking were all flagged and removed. Therefore, we were limited to targeting a geographic area (New York City Designated Market Area in this study) and basic demographics (age and sex only) for the first round of survey recruitment. Our first attempt at ad placement received over 1000 automated responses in the first hour. We quickly reformatted our ads, removing all information about survey compensation. Our second attempt was flagged by Meta as “discriminatory” because we included eligibility criteria in the ad’s text description. We removed this as well and successfully launched the first ad (Figure 1). We used 3 different ad images; we started with an image of a woman as one of the parent study research goals is to understand harm reduction engagement barriers for women. As the majority of Meta respondents were men, we used 2 additional images of women of varying races and ethnicities in an attempt to increase female participants, as men did not seem dissuaded by images of women. All images were obtained from stock photography websites offering copyright- and royalty-free images (eg, Unsplash).

In addition, we contacted several Facebook groups related to substance use and posted about the survey; once we received permission from moderators to join, all of our posts were removed by moderators and yielded no participants.

#### X (Formerly Twitter)

Purchasing ads on X is similar to Meta’s platform, where the advertiser sets a campaign goal (website traffic and conversions), a daily spending limit, and a campaign duration. Ads can also be targeted to specific demographics and geographic locations. Unlike Meta, X allows ad targeting using common keywords and hashtags. Targeted keywords included “opioid,” “addiction,” “recovery,” “opiates,” “overdose,” and “harm reduction,” as well as options for specific opioids (eg, oxycodone). However, we found that the available keywords were in flux, with some keyword options disappearing in between ad purchases. There appeared to be even less transparency in X’s algorithm regarding how cost per click is calculated compared with Meta, as the count of link clicks appeared to update haphazardly while Meta updated in real time.

**Figure 2:**
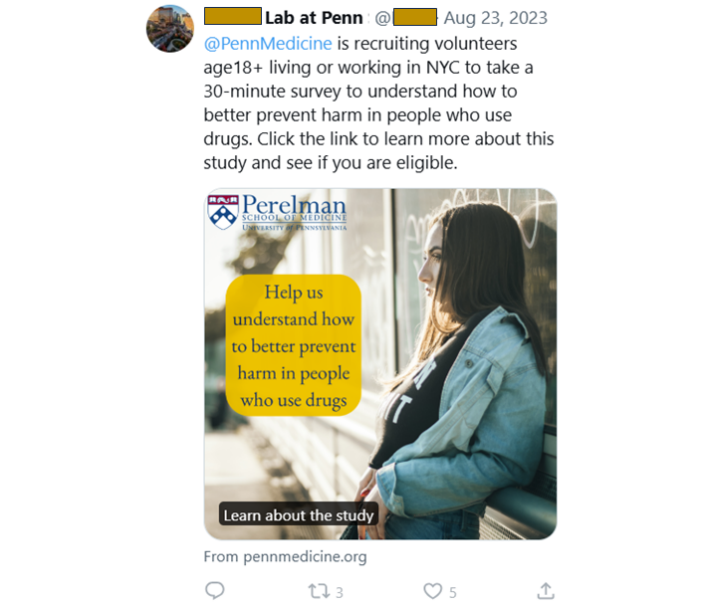
Example of an advertisement approved by X (formerly Twitter). NYC: New York City.

As with Meta, our ads on X did not contain any mention of compensation or inclusion criteria (Figure 2). Our first attempt at ad placement was flagged by X as “ineligible” because of an unspecified policy violation, and our account was banned from purchasing ads. We appealed this decision by emailing ad support and explaining that the campaign was for an academic study. A customer service bot replied that our ad violated “unacceptable business practices” by promoting “misleading, false, or unsubstantiated claims.” We replied to the email explaining the study purpose; ad support replied that they had manually reviewed the ad, they would not review the ad again, and we were permanently banned from purchasing ads. Approximately 12 hours later, we received another email from ad support alerting us they had “resolved all issues,” and our ad campaign began.

#### Reddit

**Figure 3:**
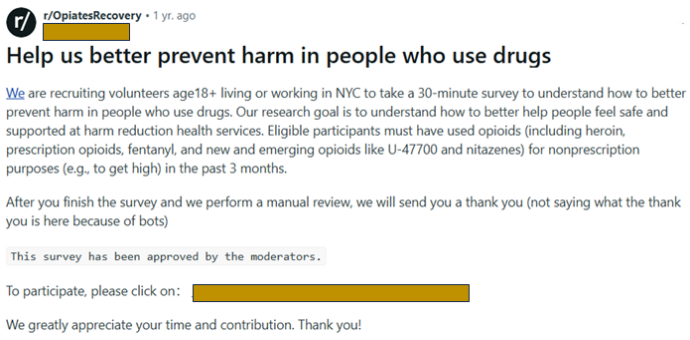
Example of a Reddit post approved by subreddit moderators. NYC: New York City.

Due to internal guidelines, our institution did not support purchasing ads on Reddit. Instead, we identified subreddits focused on topics and populations related to our study objectives (eg, /r/OpiatesRecovery) [37] and contacted moderators directly to ask for permission to recruit on the subreddit. Many subreddits explicitly banned academic study recruitment in the subreddit rules, and we did not contact those subreddits. We contacted 50 subreddits and received permission from 16 (N=4,048,000 total members; memberships are not mutually exclusive). Reddit provides engagement metrics for all user posts, so we tracked when engagement with a post waned—on average, after a week. We posted on most subreddits weekly during the recruitment period, except for 1 subreddit that specifically requested we not post more often than monthly. Study staff posted the advertisements, and they were marked, “This survey has been approved by the moderators” (Figure 3). Many substance use–related subreddits do not allow images to be posted, so subreddit posts did not include ad images. No subreddits charged for posts.

#### Discord

Launched in 2015, Discord allows users to create private or public communities called servers where members can chat in real time; servers can be subdivided into topic-based channels, which resemble message boards or subreddits. As with Reddit, Discord’s servers are monitored by administrators who enforce the server’s code of conduct. However, because Discord is not a centralized system but rather a series of servers created by individual administrators, there is less regulation from a central authority about inflammatory or illegal content such as drug selling. Individual administrators determine what is acceptable behavior on their server.

**Figure 4:**
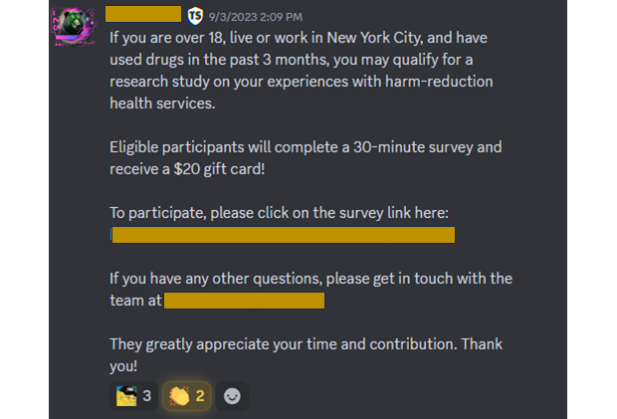
Example of a Discord post approved by server administrators.

Currently, there is no mechanism to purchase ads on Discord. We used targeted keyword searches, including “recovery,” “addiction,” and “opioids,” to identify Discord servers to contact for survey promotion. An additional server was suggested via administrator recommendation. We avoided servers with active drug dealing. Of the 14 servers approached, administrators from 7 servers granted permission for survey dissemination. Administrators from these servers posted ads on our behalf as an announcement (Figure 4). Discord does not track user analytics for individual posts, but we recorded the number of server members for each community to estimate the potential reach of our posts (N=21,582 total members; memberships are not mutually exclusive). While some individual Discord servers may charge to approve advertisement posts, we did not encounter any.

Moderators from one of the Discord channels we approached (Bluelight.org) also invited us to post on their web forum community, a public website where members can discuss and share information on drug-related topics through posted messages.

### Survey Procedures

We conducted an internet-based survey on engagement with harm reduction health services (eg, syringe exchange). Participants must be ages 18 years and older, live or work in New York City (defined as the five boroughs: Manhattan, Bronx, Brooklyn, Queens, and Staten Island), and have used opioids (including heroin, nonprescription opioids, or fentanyl) for nonprescription purposes (eg, to get “high”) in the previous 90 days. Harm reduction health services availability varies widely across the United States; therefore, we chose to limit the geographic area to New York City to better understand engagement with existing services in the city. Recruiting a sample circumscribed by a geographic area may also provide locally relevant results for public health planning purposes. Respondents completed the self-directed questionnaire in REDCap (Research Electronic Data Capture; version 13.9.3; Vanderbilt University) at a location and time of their choosing. The survey was only available in English and was designed to take around 30 minutes to complete. Data collection took place from August 2023 to November 2023.

After clicking on an advertisement, participants were directed to the study homepage, which included more information on the study’s purpose, eligibility criteria, and compensation. The study homepage was hosted on TrialX iConnect, a clinical trial recruitment platform, allowing study staff to track homepage views. This site also provides unlimited tracked hyperlinks, allowing researchers to track the number of social media users engaging with a given hyperlink. However, because of bots and repeat visitors, these proved unreliable. For example, one subreddit post that received only 533 views reported 1786 link users over the same time period.

The TrialX iConnect home page is linked to the informed consent and eligibility screening in REDCap. Participants were required to provide their age and whether they lived or worked in New York City. Each option also required the participant to select the borough where they lived or worked and provide a ZIP code unless the participant indicated they were not currently working. Questions on substances used in the past 90 days and mode of use (injecting, snorting, oral, and smoking) were adapted from the Alcohol, Smoking, and Substance Involvement Screening Test (ASSIST) [38] and expanded to include questions on newer and emerging synthetic opioids (U-47700, nitazenes) and other substances (eg, synthetic cannabinoids and xylazine). There were also several open-ended questions throughout the survey where participants were invited to provide more information about their answers (eg, “In your own words, can you describe what services you received the last time you visited a needle exchange program?”).

After completing the survey, participants were given the option to receive a US $20 gift card by email from their choice of 4 vendors (Amazon, Target, Starbucks, or Chipotle). Participants were given the option not to provide an email address and forgo the compensation or email the principal investigator directly. After gift card options, we provided a resource guide with information on support services for substance use and mental health in New York City. We also provided a link to the TrialX iConnect homepage for participants to optionally send to others; this link was changed weekly to prevent repeat responses. Several studies have shown that respondent-driven sampling, where people who use opioids refer other people who use opioids to participate, is particularly useful with hard-to-reach populations [39,40].

### Bot Prevention

REDCap includes Google’s reCAPTCHA service, a type of challenge-response test to determine whether the user is human in order to deter bot attacks and spam. However, REDCap does not allow the programmer to choose the level of CAPTCHA security; rather, Google decides the stringency of the test. In addition, we included a picture of a group of objects (eg, 4 cats) and asked respondents to enter the number of the object in a text box. This is because it is more cumbersome to program a bot to count objects in an image versus identifying an object [41]. REDCap does not allow images to randomly change in a survey, so study staff manually changed the image periodically to prevent manually programmed bots from continuing the survey. We included “honeypot” questions—items programmed to be hidden from respondents but answered by automated bots [32]—but no respondents answered these questions. We collected IP addresses to determine whether multiple surveys were submitted from the same location [42]. We also included a fictitious drug to detect participants who did not answer honestly or carefully [42]. We did not collect any personally identifiable information other than IP and email addresses for gift card distribution.

### Post Hoc Data Inspection

We implemented a rigorous, multistep post hoc data cleaning protocol to detect bot submissions, repeat participants, and other mischievous or malicious responders (see Figure S2 in Multimedia Appendix 1). First, we excluded IP addresses outside the United States or Canada and duplicate IP and email addresses. We excluded respondents who endorsed the use of a fictitious drug. We also excluded participants who reported living or working in invalid ZIP codes (eg, 10048, the retired ZIP code for the World Trade Center) or if the ZIP codes did not match the borough in which they reported living or working (eg, the participant chose “Brooklyn” from the drop-down menu but wrote in a home ZIP code in Buffalo, NY). We checked that participants who listed invalid or discrepant ZIP codes did not also report experiencing homelessness, as this might explain a missing or invalid ZIP code. We next eliminated records reporting improbable or impossible drug use behaviors (eg, injecting alcohol, injecting inhalants, or injecting marijuana), as well as records reporting more than 10 lifetime overdoses. Data inspection showed some respondents listed as many as 105 lifetime overdoses. A New York City study using respondent-driven sampling for in-person data collection found that, while self-reported lifetime overdoses increased with the COVID-19 pandemic, median lifetime overdoses was 3 (IQR 2-6), and only 3.6% (n=10) of respondents reported 10 or more lifetime overdoses [43].

We used the Density-Based Spatial Clustering of Applications with Noise (DBSCAN) algorithm to identify clusters of behavior where a single individual completes the survey multiple times with the same response set. The DBSCAN algorithm is particularly useful because clusters can be of arbitrary size, shape, and number [44]. We used the DBSCAN function in the *Scikit-learn* package for Python (version 3.10.10; Python Software Foundation) [45-47]. Not all variables could be included because the algorithm requires complete records; therefore, variables that could be left blank could not be used in the analysis. We specifically targeted demographics (eg, sex, race and ethnicity) and variables related to drug use frequency and mode of use. We limited the algorithm to a very narrow cluster radius, so records must be nearly identical to be flagged as part of a cluster.

After eliminating records flagged for repeated entries, we excluded all records completed in under 7 minutes as this was below the fastest time study staff completed the survey during pilot testing [32,48]. We also examined demographic inconsistencies (eg, Medicaid health insurance and household income over eligibility levels). We then eliminated records with suspicious responses to open-ended questions. These responses often did not correspond to the question or appeared to be AI-generated. For example, when asked to describe the types of medical services received at a needle exchange program, eliminated responses included “very friendly informative and caring,” “quite encouraging and satisfying,” “it’s good,” and “A magazine gauidiance [sic].” We also excluded records with identical or near-identical odd phrasing for these responses (eg, “General body check up [sic]” for the medical services question). As a final check, we eliminated records with email addresses comprised of random letters ending in numbers exceeding 4 digits, as these characteristics are an indication of bulk email account creators that can be built or purchased [32,49].

### Data Analysis

We present basic frequencies to provide a description of the included data. We also performed a supplemental analysis of excluded records to identify any possible patterns or significant differences from the included data. We compared excluded versus included data using the Pearson chi-square test of independence for categorical variables or the Welch sample *t* test for ratio variables.

### Ethical Considerations

This research was approved by the institutional review board at the University of Pennsylvania (protocol 853254). All procedures performed were in accordance with the ethical standards of the University of Pennsylvania institutional review board and with the 1964 Helsinki Declaration and its later amendments or comparable ethical standards. Written informed consent was obtained from all participants before they began the survey.

## Results

We collected a total of 2560 complete records, 93.2% (n=2387) of which were determined to be from bots or bad actors. The majority of included responses (n=173) were collected from Meta (n=79, 45.7%) and Reddit (n=48, 27.8%; see Table 1). Meta’s ads cost US $0.68/click, or US $14.60 per complete record. X’s ads were the most expensive (US $1.96/click) and yielded the fewest participants (3 completed surveys). This resulted in a cost of US $178.33 per completed record for participants recruited through X. We recruited 22.5% (n=39) of the sample through participant peer referral. Overall advertising cost was US $9.76 per participant in the final sample.

Post hoc data investigation found that 23.9% (571/2560) of excluded records showed a duplicate IP or email address, 45.9% (1095/2560) reported consuming a fictitious drug, 15.8% (378//2560) provided an invalid ZIP code, and 9.8% (235/2560) reported improbable drug use behaviors (Table 2). The largest DBSCAN cluster contained 42 records. We cut off clusters of interest at clusters containing 6 or more records (222/2560, 8.7%) and then analyzed the individual records, noting those that appeared within a short time frame of each other. Overall, 13.7% (351/2560) of all records were in clusters and excluded. Figure S1 in Multimedia Appendix 1 presents a description of how many records were excluded at each step of post hoc data management to arrive at the final sample.

The mean age of the final sample was 29.9 years (SD 6.5; range 18-63 years; see Table 3). A plurality of the sample was male (102/173, 59%) and non-Hispanic Black (77/173, 44.5%). Approximately half of the sample reported using prescription opioids in the previous 90 days (144/173, 51.8%), while 24% (67/173) reported fentanyl use and 20% (54/173) reported heroin use. Oral consumption was the most common mode of use reported (143/173, 83.7%), followed by injection (59/173, 34.1%). A majority of the sample also reported using alcohol (133/173, 76.9%) and marijuana (90/173, 52%) in the previous 90 days. Other commonly used substances included benzodiazepines (51/173, 29.5%) and 3,4-methylenedioxymethamphetamine (MDMA) (36/173, 20.8%). One-fifth reported no lifetime experience with harm reduction services (34/173, 19.7%), and 10.4% (18/173) reported receipt of condoms as their only lifetime harm reduction experience; 40.5% (70/173) reported no harm reduction engagement in the previous 90 days and 7.5% (13/173) reported receipt of condoms as their only engagement in the previous 90 days. Receipt of condoms was the most commonly reported type of lifetime experience with harm reduction services (79/173, 45.7%), followed by receipt of naloxone (66/173, 38.2%). Receiving sterile syringes from a pharmacy was slightly more common than from a needle exchange program (Lifetime: 49/173, 28.3% vs 41/173, 23.7%; *P*=.33; Past 90 days: 26/173, 15% vs 25/173, 14.5%; *P*=.87).

In the supplemental analysis of excluded records, 49% (n=1169) reported they were non-Hispanic white and 44.5% (n=1062) reported non-Hispanic Black, with significantly fewer reporting being Hispanic or other races or ethnicities than included records (*P*<.001; see Table S1 in Multimedia Appendix 1). A larger percentage of excluded records reported to be men (compared with women), a significantly greater difference compared with the included responses (*P*=.002). Excluded records also reported lower levels of education (*P*<.001) and higher levels of income (*P*<.001) compared with included responses. Excluded records had significantly higher reports of heroin and fentanyl use than included records (*P*<.001), as well as significantly higher use of all other substances (*P*<.001). Excluded records also showed significantly more smoking and snorting of opioids and significantly less oral consumption (*P*<.001). Excluded records reported significantly more lifetime overdoses compared with included records (included: mean 1.0, SD 1.51; excluded: mean 4.3, SD 9.43; *P*<.001). There were no significant differences in age, sexual orientation, or housing stability.

**Table 1:** Recruitment number and cost by social media platform, omitting records excluded during post hoc data management.

Platform	Impressions	Clicks, n	Cost/click (US $)	Total cost (US $)	n (%)	Cost/N (US $)
Meta (Facebook and Instagram)	165,751	1700	0.68	1153.26	79 (45.7)	14.60
X (formerly Twitter)	161,792	270	1.96	535	3 (1.7)	178.33
Reddit	50,508	—^a^	0	0	48 (27.8)	0
Discord	—	—	0	0	3 (1.7)	0
Bluelight web forum	—	—	0	0	1 (0.6)	0
Friend referral	—	—	—	—	39 (22.5)	0

^a^Not available.

**Table 2:** Post hoc data management violations (N=2560 total complete records).

Violation category	Total records, n (%)
IP addresses outside the United States or Canada	279 (10.9)
Duplicate IP and, or email addresses	571 (22.3)
Endorsed use of fictitious drug	1095 (42.8)
Invalid ZIP codes (eg, 10048)	400 (15.6)
ZIP codes not in the reported borough	124 (4.8)
Improbable drug use behaviors (eg, injecting alcohol, injecting marijuana)	235 (9.2)
>10 lifetime overdoses	232 (9.1)
Clustering (DBSCAN^a^)	351 (13.7)
<7-minute survey response times	76 (3)
Demographic discrepancies (eg, income and insurance)	32 (1.3)
Suspicious responses to open-ended questions (eg, “A magazine gauidiance” for medical services)	116 (4.5)
Email address check	15 (0.6)

^a^DBSCAN: Density-Based Spatial Clustering of Applications with Noise.

**Table 3:** Description of the sample after post hoc data inspection (N=173).

Variable				Total (N=173)
Age (years), mean (SD)				29.86 (6.52)
**Gender, n (%)**				
	Man			102 (58.96)
	Woman			62 (35.84)
	Transgender			4 (2.31)
	Nonbinary			4 (2.31)
	Prefer not to say			1 (0.58)
**Race and ethnicity, n (%)**				
	Non-Hispanic White			68 (39.31)
	Non-Hispanic Black			77 (44.51)
	Hispanic			13 (7.51)
	Asian			7 (4.05)
	Mixed race			6 (3.47)
	Other			2 (1.16)
**Sexual orientation, n (%)**				
	Heterosexual			141 (81.50)
	Bisexual			18 (10.40)
	Homosexual or queer			11 (6.36)
	Prefer not to say			3 (1.73)
**Home borough, n (%)**				
	Manhattan			39 (23.08)
	Brooklyn			57 (33.73)
	Bronx			29 (17.16)
	Queens			26 (15.38)
	Staten Island			12 (7.10)
	Outside of New York City			6 (3.55)
**History of homelessness, n (%)**				
	Stably housed, no past homelessness			102 (58.96)
	Stably house, past homelessness			46 (26.59)
	Current homelessness			15 (8.67)
	Stably housed but worried about housing			10 (5.78)
**Income (US $), n (%)**				
	0-29,999			34 (19.65)
	30,000-39,999			26 (15.03)
	40,000-49,999			9 (5.2)
	50,000-59,999			22 (12.72)
	60,000-74,999			20 (11.56)
	75,000-99,999			36 (20.81)
	100,000 or more			19 (10.98)
	Don’t know			7 (4.05)
**Education level, n (%)**				
	Less than a high school diploma			2 (1.16)
	A high school diploma or General Educational Development (GED) test			21 (12.14)
	Some college or a 2-year degree			59 (34.10)
	4-year college degree			74 (42.77)
	Postgraduate work			17 (9.83)
**Health insurance, n (%)**				
	Yes			136 (78.61)
	No			41 (23.70)
**Children, n (%)^a^**				
	Living with biological child <18 years old			37 (21.39)
	Not living with child <18 years old (eg, child with grandparent)			19 (10.98)
	No children or no children <18 years old			104 (60.12)
**Opioid use, past 90 days, n (%)^a^**				
	Prescription			144 (51.80)
	Heroin			54 (19.42)
	Fentanyl			67 (24.10)
	Other			13 (4.68)
**Mode of opioid use, n (%)^a^**				
	**Oral, total**			143 (82.66)
		Prescription	125 (70.22)	
		Heroin	3 (4.69)	
		Fentanyl	28 (35.44)	
		Other	9 (69.23)	
	**Snort, total**			34 (19.65)
		Prescription	14 (7.87)	
		Heroin	18 (28.13)	
		Fentanyl	16 (20.25)	
		Other	1 (7.69)	
	**Smoke, total**			21 (12.14)
		Prescription	6 (3.37)	
		Heroin	9 (14.06)	
		Fentanyl	9 (11.39)	
		Other	1 (7.69)	
	**Inject, total**			59 (34.10)
		Prescription	33 (18.54)	
		Heroin	34 (53.13)	
		Fentanyl	26 (32.91)	
		Other	2 (15.38)	
**Other substances, past 90 days, n (%)^a^**				
	Alcohol			133 (76.88)
	Marijuana			90 (52.02)
	Cocaine			33 (19.08)
	Benzodiazepines			51 (29.48)
	Methamphetamine			22 (12.72)
	3,4-Methylenedioxymethamphetamine (MDMA)			36 (20.81)
	Ketamine			17 (9.83)
	Amphetamines (eg, Adderall)			34 (19.65)
	Hallucinogens			18 (10.40)
	Synthetic marijuana (K2, spice)			26 (15.03)
	Xylazine			10 (5.78)
	Inhalants			12 (6.94)
**Harm reduction experience (lifetime), n (%)^a^**				
	Needle exchange program for needles			41 (23.70)
	Needle exchange program for health services			24 (13.87)
	Needles from pharmacy			49 (28.32)
	Naloxone (Narcan)			66 (38.15)
	Sterile works kit			36 (20.81)
	Safer smoking items (eg, pipes and stems)			30 (17.34)
	Drug testing (eg, fentanyl test strips)			47 (27.17)
	Pre-exposure prophylaxis (PrEP) or postexposure prophylaxis (PEP)			30 (17.34)
	Condoms			79 (45.66)
	Visit an overdose prevention center or supervised consumption site			41 (23.70)
**Harm reduction experience (past 90 days), n (%)^a^**				
	Needle exchange program for needles			25 (14.45)
	Needle exchange program for health services			15 (8.67)
	Needles from pharmacy			26 (15.03)
	Naloxone (Narcan)			47 (27.17)
	Sterile works kit			22 (12.72)
	Safer smoking items (eg, pipes and stems)			20 (11.56)
	Drug testing (eg, fentanyl test strips)			31 (17.92)
	Pre-exposure prophylaxis (PrEP) or postexposure prophylaxis (PEP)			18 (10.4)
	Condoms			54 (31.21)
	Visit an overdose prevention center or supervised consumption site			20 (11.56)
**Lifetime history of overdose, mean (SD)**				1.00 (1.51)
	Zero			98 (56.65)
	≥1			75 (43.35)
**Depression (Center of Epidemiologic Studies Depression Scale-10), mean (SD)**				11.4 (5.4)
	Depressed (≥10)			106 (61.3)
	Not depressed (<10)			67 (38.7)
**Anxiety (General Anxiety Disorder-7), mean (SD)**				6.9 (5.3)
	Minimal anxiety (0-4)			69 (39.88)
	Mild anxiety (5-9)			49 (28.32)
	Moderate anxiety (10-14)			45 (26.01)
	Severe anxiety (≥15)			10 (5.78)

^a^Categories are not mutually exclusive.

## Discussion

### Principal Findings

Our experience provided an opportunity to assess the continued viability of social media platforms for recruiting a hard-to-reach population of people who use opioids. While we successfully collected final survey data from a diverse sample of 173 people who use opioids, the study was not without challenges. Even the first step—setting up advertisements across platforms—proved difficult. Most institutional review boards require recruitment advertisements to contain detailed information on study purpose, compensation, and inclusion criteria, among other elements, but we found that including these details negatively affected our ability to use these platforms for recruitment. Including compensation information in advertisements resulted in an almost immediate influx of bots on Meta. Meta also flagged our ads as “discriminatory” when we included inclusion criteria, and X (formerly known as Twitter) flagged our study as “spreading false information” when we included study purposes. Therefore, we had to weigh best practices for ethical recruitment with social media platform policies and bot avoidance.

Our recruitment results are consistent with previous studies of social media recruitment, which found the most success with social media recruitment on Meta’s platforms (n=79, 45.7%) [50]. In our study, Meta’s ads cost US $0.68/click, or US $14.60 per complete record. This is an increase from a 2017 systematic review of 35 studies, which found a median cost of US $0.51 per click and cost per participant of US $14.41 for Facebook ads [12]. We found recruitment on X to be expensive for low enrollment despite the ability to use tailored keywords to better target ads. We only recruited 3 participants through X at a cost of US $1.96 per click and US $178.33 per participant. A few previous surveys used paid advertisements on Twitter to recruit participants for substance use studies; our results are comparable with previous studies, which found that recruitment through Twitter was high cost with little or no recruitment [50,51]. While Reddit and Discord recruitment posts were unpaid, the time and resources devoted to identifying and contacting moderators for individual subreddits and servers, posting ads, and monitoring comments were cumbersome for the number of yielded participants (Reddit: n=48, 27.8%; Discord: n=3, 1.7%).

With respect to post hoc data investigation, we determined that 93% (2387/2560) of collected records were invalid or suspicious despite using REDCap’s built-in reCAPTCHA for bot prevention. Excluded records reported significantly more heroin and fentanyl use than included records, as well as significantly more use of all other substances. While previous studies of people who use opioids using social media recruitment have used screening protocols to verify participants [18,42], the proliferation of bots and malicious responders has made it increasingly difficult to verify participant eligibility and data validity. IP addresses are not consistently reliable in identifying repeat participants because IP addresses change periodically at unknown intervals, and IP addresses can be reset manually. Furthermore, IP address location is not reliable for determining geographic eligibility because the IP address location may list the headquarters of the internet provider and not necessarily where the device accessing the internet is located [52]. Virtual private networks (VPNs), which ensure a secure encrypted connection between the user and the internet, have become considerably cheaper in recent years and can be used to artificially change the geographic location of the user’s IP address. Future studies should investigate new techniques for tracking website visitors, most of which have not yet been incorporated into available internet-based survey platforms to our knowledge. Urchin Tracking Module (UTM) codes are used by Google Analytics to track the behavior of website visitors. They are often used to track which channels generate the most website traffic for commercial purposes. UTM codes have been successfully used to track participant recruitment to health studies from various online forums, including Facebook ads [53], though to our knowledge, they have not been applied to other social media platforms. They might be useful for tracking recruitment across social media platforms and to identify repeat participants. More advanced tracking systems, such as recording media access control (MAC) address—the unique number assigned to each device connected to a network—may also be useful to better identify repeat participants. MAC identifiers have been used in previous health studies but not to identify repeat survey participants to our knowledge [54,55]. In the absence of more advanced technologies to weed out fraudulent responses, researchers should continue to include data validity tests (eg, a fictitious drug and open-ended qualitative response questions). Bot-targeted screeners such as reCAPTCHA and honeypot questions are insufficient as individual human fraudsters become more common [31]. As the purpose of bots and other fraudulent responders is to closely mimic valid data for monetary compensation, researchers must remain vigilant against how stigma and other biases may impact their expectations regarding people who use opioids’ reported behavior [56].

### Limitations

This study was a cross-sectional internet-based survey using a convenience sample of people who use opioids in one large US metropolitan area. It is possible that social media platform preference and frequency of use differ in other US cities or globally. Due to institutional regulations, we were unable to purchase ads on Reddit or TikTok. There is a possibility of selection bias resulting from which subreddits, Discord servers, and social media platforms agreed to allow recruitment. We spoke with a research advancement manager at Meta who suggested using the participant data collected to create a “lookalike” audience, or a group of participants representing the ideal survey participant. We were unable to do this because emails provided by the participants to receive the survey gift card did not match emails from registered Facebook users. This may have limited our ability to effectively target our Meta ads to people who use opioids. As this study aimed to engage people who use opioids unaffiliated with community-based organizations to study barriers to harm reduction services engagement, we were not able to partner with community-based organizations for recruitment; study recruitment materials may have benefited from input from people who use opioids with lived experiences. As the iConnect links were unable to effectively track traffic from different social media platforms, we relied on self-reporting to identify where participants found the survey. While REDCap includes Google’s reCAPTCHA service to deter bot attacks and spam, REDCap does not allow the programmer to choose the level of CAPTCHA security. Other internet-based survey platforms may include more intense protection or allow the user to decide the stringency of the CAPTCHA test, reducing the reliance on post hoc data management [32]. While we developed a rigorous post hoc data cleaning protocol, it is possible that invalid data were included in our study while valid surveys were excluded. We may have incorrectly excluded records based on IP address location if respondents used a VPN or Tor browser to hide their IP address due to concerns about being tracked. Due to the stigmatized nature of drug use, and people who use opioids’ legitimate concerns of legal implications for admitting to illegal behaviors, we limited our collection of personal identifiable information (PII) to the collection of IP and email addresses. Other internet-based health surveys have been able to compare names and home addresses against verified national databases to eliminate invalid records [57]. Future internet-based research on opioid use may need to balance the ethics of collecting PII with potentially including invalid data.

### Conclusion

Social media recruitment of hidden populations is challenging but not impossible. Rigorous data collection protocols and post hoc data management are necessary to ensure the validity of findings; these methods may counter previous best practices for researching stigmatized behaviors.

## Appendix

Multimedia Appendix 1 Further description of excluded records by post hoc data management step and comparison of included versus excluded records.

## Abbreviations

API: application programming interface
ASSIST: Alcohol, Smoking, and Substance Involvement Screening Test
DBSCAN: Density-Based Spatial Clustering of Applications with Noise
MAC: media access control
MDMA: 3,4-methylenedioxymethamphetamine
NYC: New York City
PII: personal identifiable information
REDCap: Research Electronic Data Capture
UTM: Urchin Tracking Module
VPN: virtual private network
