# Supplementary material for: Challenging the Continued Usefulness of Social Media Recruitment for Surveys of Hidden Populations of People Who Use Opioids
Source: J Med Internet Res. 2025 Apr 30;27:e63687. doi: 10.2196/63687 (PMC12079069; doi:10.2196/63687)
Supplement: Multimedia Appendix 1 [file jmir_v27i1e63687_app1.docx]

**Challenging the continued usefulness of social media recruitment for surveys of hidden populations of people who use opioids**

Elizabeth D. Nesoff, PhD MPH; Joseph J. Palamar, PhD; Qingyue Li, MSSP;

Wenqian Li, MSSP; Silvia S. Martins, MD PhD

**Supplemental Material**

**Figure S1**. Excluded records by post hoc data inspection step.


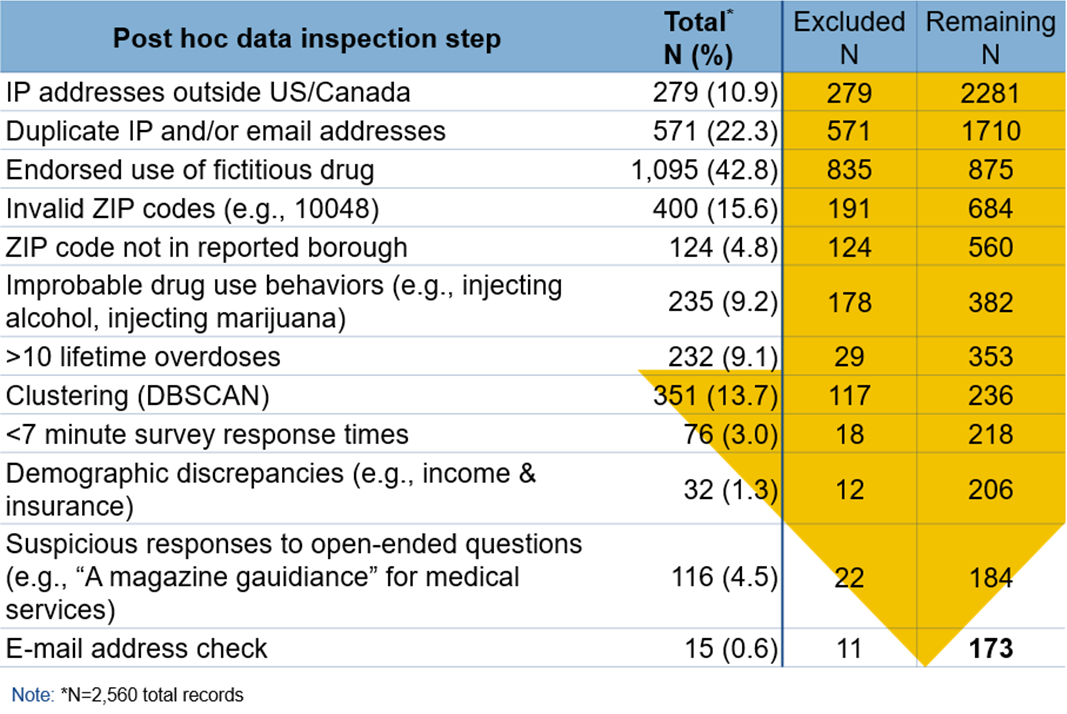


**Table S1**. Comparison of included and excluded records.

| **Variable** | **Included (n=173)** | **Excluded**  **(n=2387)** | ***P***^ǂ^ |
| --- | --- | --- | --- |
| Age, mean (sd) | 29.86 ± 6.52 | 30.19±7.03 | .550 |
| Gender n (%)  Man  Woman  Transgender  Non-binary  Prefer not to say | 102 (58.96)  62 (35.84)  4 (2.31)  4 (2.31)  1 (0.58) | 1655 (69.33)  689 (28.86)  29 (1.21)  9 (0.38)  5 (0.21) | .002 |
| Race/ethnicity, n (%)  Non-Hispanic white  Non-Hispanic Black  Hispanic  Other | 68 (39.31)  77 (44.51)  13 (7.51)  15 (8.68) | 1169 (48.97)  1062 (44.49)  69 (2.89)  87 (3.64) | <.001 |
| Sexual orientation, n (%)  Heterosexual  Bisexual  Homosexual/Queer  Other  Prefer not to say | 141 (81.50)  18 (10.40)  11 (6.36)  0  3 (1.73) | 1862 (78.01)  256 (10.72)  203 (8.5)  41 (1.72)  25 (1.05) | .337 |
| Home borough, n (%)  Manhattan  Brooklyn  Bronx  Queens  Staten Island  Outside of NYC | 39 (23.08)  57 (33.73)  29 (17.16)  26 (15.38)  12 (7.10)  6 (3.55) | 760 (32.07)  635 (26.79)  407 (17.17)  379 (15.99)  169 (7.13)  20 (0.84) | .003 |
| History of homelessness, n (%)  Stably-housed, no past homelessness  Stably-housed, past homelessness  Current homelessness  Stably-housed but worried about housing | 102 (58.96)  46 (26.59)  15 (8.67)  10 (5.78) | 1438 (60.24)  564 (23.63)  210 (8.80)  159 (6.66) | .845 |
| Income, n (%)  0 to $29,999  $30,000 to $39,999  $40,000 to $49,999  $50,000 to $59,999  $60,000 to $74,999  $75,000 to $99,999  $100,000 or more  Don’t know | 34 (19.65)  26 (15.03)  9 (5.2)  22 (12.72)  20 (11.56)  36 (20.81)  19 (10.98)  7 (4.05) | 546 (22.87)  244 (10.22)  152 (6.37)  235 (9.84)  215 (9.01)  435 (18.22)  542 (22.71)  14 (0.59) | <.001 |
| Education level, n (%)  No formal schooling  Less than a high school diploma  A high school diploma or GED  Some college or a 2-year degree  4-year college degree  Post-graduate work | 0 (0.0)  2 (1.16)  21 (12.14)  59 (34.10)  74 (42.77)  17 (9.83) | 37 (1.55)  99 (4.15)  347 (14.56)  472 (19.81)  1009 (42.34)  419 (17.58) | <.001 |
| Opioid use, past 90 days, n (%)^*^  Prescription  Heroin  Fentanyl  Other | 144 (83.24)  54 (31.21)  67 (38.73)  13 (7.51) | 2012 (84.29)  1467 (61.46)  1244 (52.12)  658 (27.57) | <.001 |
| Mode of opioid use, past 90 days, n (%)^*^  Oral  Snort  Smoke  Inject | 143 (82.66)  34 (19.65)  21 (12.14)  59 (34.10) | 1729 (72.43)  899 (37.66)  730 (30.58)  868 (36.36) | <.001 |
| Other substances, past 90 days, n (%)^*^  Alcohol  Marijuana  Cocaine  Benzodiazepines  Methamphetamine  MDMA  Ketamine  Amphetamines (e.g., Adderall)  Hallucinogens  Synthetic Marijuana (K2, spice)  Xylazine  Inhalants | 133 (76.88)  90 (52.02)  33 (19.08)  51 (29.48)  22 (12.72)  36 (20.81)  17 (9.83)  34 (19.65)  18 (10.40)  26 (15.03)  10 (5.78)  12 (6.94) | 1951 (81.73)  1469 (61.54)  1128 (47.26)  915 (38.33)  962 (40.30)  958 (40.13)  928 (38.88)  1000 (41.89)  900 (37.70)  978 (40.97)  771 (32.30)  916 (38.37) | <.001 |
| Lifetime history of overdose, mean (sd)  Zero  ≥1 | 1.00 (1.51)  98 (56.65)  75 (43.35) | 4.30 (9.43)  940 (39.38)  1447 (60.62) | <.001  <.001 |
| Social media recruitment platform, n (%)  Meta (Facebook)  X (Twitter)  Reddit  Discord  Friend referral | 79 (45.7)  3 (1.7)  48 (27.8)  4 (2.3)  39 (22.5) | 981 (41.10)  917 (38.42)  329 (13.78)  121 (5.07)  39 (1.63) | <.001 |

^*^Categories are not mutually exclusive

^ǂ^Pearson chi-square test of independence for categorical variables or Welch two sample t-test for continuous variables
